# Consuming More of Daily Caloric Intake at Dinner Predisposes to Obesity. A 6-Year Population-Based Prospective Cohort Study

**DOI:** 10.1371/journal.pone.0108467

**Published:** 2014-09-24

**Authors:** Simona Bo, Giovanni Musso, Guglielmo Beccuti, Maurizio Fadda, Debora Fedele, Roberto Gambino, Luigi Gentile, Marilena Durazzo, Ezio Ghigo, Maurizio Cassader

**Affiliations:** 1 Department of Medical Sciences, University of Turin, Turin, Italy; 2 Emergency Department, Gradenigo Hospital, Turin, Italy; 3 Dietology Unit, Città della Salute e della Scienza, Turin, Italy; 4 Diabetic Clinic, Hospital of Asti, Asti, Italy; Institut d’Investigacions Biomèdiques August Pi i Sunyer, Spain

## Abstract

**Background/Objectives:**

It has been hypothesized that assuming most of the caloric intake later in the day leads to metabolic disadvantages, but few studies are available on this topic. Aim of our study was to prospectively examine whether eating more of the daily caloric intake at dinner leads to an increased risk of obesity, hyperglycemia, metabolic syndrome, and non-alcoholic fatty liver disease (NAFLD).

**Subjects/Methods:**

1245 non-obese, non-diabetic middle-aged adults from a population-based cohort underwent a 3-day food record questionnaire at enrollment. Anthropometric values, blood pressure, blood metabolic variables, and estimated liver fat were measured at baseline and at 6-year follow-up.

**Design:**

Prospective cohort study.

**Results:**

Subjects were divided according to tertiles of percent daily caloric intake at dinner. A significant increase in the incidence rate of obesity (from 4.7 to 11.4%), metabolic syndrome (from 11.1 to 16.1%), and estimated NAFLD (from 16.5 to 23.8%) was observed from the lower to higher tertile. In a multiple logistic regression model adjusted for multiple covariates, subjects in the highest tertile showed an increased risk of developing obesity (OR = 2.33; 95% CI 1.17–4.65; p = 0.02), metabolic syndrome (OR = 1.52; 95% CI 1.01–2.30; p = 0.04), and NAFLD (OR = 1.56; 95% CI 1.10–2.22; p = 0.01).

**Conclusions:**

Consuming more of the daily energy intake at dinner is associated with an increased risk of obesity, metabolic syndrome, and NAFLD.

## Introduction

A novel, intriguing hypothesis suggests it is not what you eat, but when you eat that plays a role in weight balance. Breakfast skipping has been associated with weight gain and obesity, dyslipidemia, diabetes and cardiovascular diseases in adults [1]–[4]. Breakfast skippers usually make less healthy food choices compared to breakfast consumers and overcompensate the intake of energy during the day [5]. However, a recent study did not confirm these findings [6]. The current trend for adults is to eat very little in the morning and shift most of the caloric intake later in the day [7]. Emerging evidence shows a relationship between the timing of food intake during the day and weight regulation in animals [8]–[10]. In humans, shifting food ingestion toward the night has been shown to disrupt metabolic homeostasis and raise postprandial triglycerides [11]. Furthermore, two cross-sectional studies in adults found an increased risk of overweight and obesity when more of the daily caloric intake was eaten in the evening [12]–[13], but a prospective cohort study found no association between evening eating and weight change [14]. Yet, late-night eaters showed an increased coronary heart disease risk in a prospective US cohort [4]. No human studies have investigated the relationship between the time of eating and non-alcoholic fatty liver disease (NAFLD), the excessive liver fat accumulation closely associated with obesity and insulin resistance, that can be predicted by noninvasive tools [15].

Our aim was to prospectively assess whether assuming more of the daily caloric intake at dinner predisposes to obesity, hyperglycemia, metabolic syndrome, and estimated liver fat content in a population-based cohort of middle-aged adults.

## Subjects and Methods

### Participants and setting

All the 45–64 years old Caucasian patients (*n* = 1877) of six general practitioners were invited to participate in a metabolic screening in 2001–2003. These subjects were representative of the age-corresponding population living in the province of Asti (Northwest Italy) [16]. Of these, 1658 (88.3%) subjects provided written informed consent to participate while 219 declined. Both the participants and non-participants were similar to the resident population of the corresponding age range in terms of male prevalence, education, prevalence of known diabetes, and residence in a rural area [16]. Patients with obesity (*n* = 315) or type 2 diabetes (*n* = 94) at baseline, and those who died during the follow-up (*n* = 61) were excluded. Since these conditions often coexisted in the same individuals, 413 patients were excluded, and data from 1245 subjects were finally analyzed.

The study was approved by the local ethics committee (“Comitato Etico Interaziendale A.S.O. SS.Antonio e Biagio e C.Arrigo” of Alessandria). All the procedures conformed to the principles of the Helsinki Declaration.

### Measurements

In the morning, after at least 12 h of fasting, weight, height, waist circumference, and blood pressure were measured. Waist circumference was measured by a plastic tape meter at the level of the umbilicus. Two blood pressure measurements were performed using mercury sphygmomanometers and appropriate cuff sizes after a 10-minute rest in the sitting position; reported values are the means of the two measurements. A blood sample was drawn for the determination of glucose, insulin, total cholesterol, HDL-cholesterol, triglycerides, alanine aminotransferase (ALT), γ-glutamyl transferase (GGT) and high-sensitivity C-reactive protein (CRP) values. If serum glucose was ≥110 mg/dl, a second fasting glucose determination was performed.

All patients were submitted to a health screening questionnaire at baseline, from July 2001 to September 2003. Data on smoking habits, alcohol consumption, educational level, health conditions, current medications (in particular drugs influencing glucose control and/or body weight, such as hypoglycemic drugs, insulin, antidepressant/antipsychotic, and estrogen/steroid use), mean weekly number of meals consumed in restaurants (including fast-food restaurants and pizzerias), and hours of sleep were collected for each subject. Sleep duration was defined as self-reported time in bed (calculated from bedtime to get up time) minus sleep latency. At baseline, patients completed the Minnesota Leisure Time Physical Activity questionnaire [17], previously validated in an European cohort [18], and the semi-quantitative food-frequency questionnaire used in the EPIC (European Prospective Investigation into Cancer and Nutrition) study [19]. The EPIC questionnaire assessed the average frequency and portion intake of 148 foods consumed in the 12 months prior to examination, but it did not evaluate the food distribution during the day. The frequency of food intake was assessed using ten categories, ranging from “never” to “five times per day or more”, whereas quantity was determined comparatively using photographs of standard portion sizes. Contemporarily, at baseline, all subjects were submitted to a 3-day food record, which consisted of a detailed written food diary. Subjects were instructed to record everything they ate or drank during 2 consecutive week days and 1 week-end day [20]. The 53 food photos and measuring guides (cups, spoons, glasses, etc) of the EPIC questionnaire aided respondents in estimating the amount of foods or beverages consumed during the compilation of the 3-day food record. The temporal pattern of food consumption was also recorded, as participants were asked to list foods eaten at breakfast, lunch, dinner, and during mid-morning, mid-afternoon, and after dinner. An instruction sheet defining each meal was given together with the food record: breakfast was defined as the meal consumed after waking up; the mid-morning meal as the food consumed after breakfast and before lunch in the morning (until 12 am), lunch as the meal consumed from 12 am to 3 pm, mid-afternoon meal as the food consumed after lunch before dinner, until 7 pm; dinner as the meal consumed from 7 pm to 10 pm; after-dinner meal as the food consumed after dinner until going to sleep. This was in line with the Italian habits.

We considered as dinner the eating occasion self-reported as dinner; skipping breakfast was defined when no consumption of any food for breakfast was reported.

When evaluating the frequency of eating occasions, we considered as eating occasion every episode with a caloric intake corresponding at least to the 15% of the total daily caloric intake [21].

A dietician, blinded to the study details, checked all questionnaires for completeness, internal coherence, and plausibility. In case of uncertain answers, the patients were interviewed by the dietician. Overall, patient compliance was high, because of the collaboration with general practitioners, who supported the patients during the recall and collection of data.

The 3-day food record data were loaded on the Win Food Pro 3 software (Medimatica, Colonnella, Teramo, Italy), and the mean nutritional values for the 3 days were reported. The reliability of the reported energy intake was assessed by calculating the ratio of estimated energy intake to predicted basal metabolic rate using age- and sex-specific formulas derived by Schofield [22]. Subjects with a ratio <0.88 were classified as under-reporters [23].

The entire sample (*n* = 1658) was then divided into three tertiles, according to percentage of daily caloric intake at dinner (the first tertile had the lowest dinner caloric intake, <33% of daily kcal; the third tertile the highest, ≥48% of daily kcal). These cutoffs divided the 1245 subjects evaluated in the final analysis into three groups of 423, 418, and 404 individuals, respectively.

The physical activity level was calculated as the product of the duration and frequency of each activity (in hours/week), weighted by an estimate of the metabolic equivalent of the activity (METS) and summed for the activities performed.

The laboratory methods have been described previously [16], [24]. Glycated hemoglobin (HbA1c) values were evaluated by High Performance Liquid Cromatography (Tosoh, Turin, Italy). This is a standardized method, as certified by the International Federation of Clinical Chemistry and Laboratory Medicine. The correlation coefficient and coefficient of variation were, respectively, 0.9998 and 0.21%.

All samples were run blindly.

### Follow-up

From January to November 2008, patients were contacted for follow-up visits. Weight, waist circumference and blood pressure were measured, and a blood sample was drawn for the determination of the same fasting metabolic parameters evaluated at baseline (see above). Since the visits were performed in collaboration with the general practitioners of the patients, we were able to contact all the patients.

### Definitions

The homeostasis model assessment of insulin resistance (HOMA-IR) [25] was employed to estimate insulin resistance. Diabetes and impaired fasting glucose (IFG) were defined according to published recommendations [26]. In particular, a diagnosis of diabetes was made in the presence of fasting plasma glucose ≥126 mg/dl; only 3 patients presented the classic symptoms of hyperglycemia and a random plasma values ≥200 mg/dl. In line with the Harmonization definition, metabolic syndrome was defined by the presence of three of the following five components: waist circumference ≥94 cm (men) or ≥80 cm (women), triglycerides ≥150 mg/dl, HDL cholesterol <40.0 mg/dl (men) or <50.0 mg/dl (women), systolic blood pressure ≥130 mmHg and/or diastolic blood pressure ≥85 mmHg and/or antihypertensive drug therapy and fasting glucose ≥100 mg/dl or hypoglycemic therapy [27]. NAFLD was estimated with the NAFLD liver fat score, according to the following formula [15]:
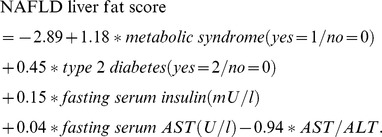



The estimated liver fat percentage was calculated with the liver fat [15]:
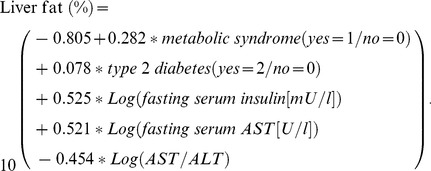



NAFLD was defined as >5% estimated liver fat percentage [15].

### Statistical analyses

Because the distributions of alcohol intake, triglyceride, fasting insulin, HOMA-IR, ALT, GGT, CRP values, and NAFLD score were highly skewed, their values were log-transformed to obtain a normal distribution. In all analyses, the log-transformed values were then used. For ease of data interpretation, the untransformed values are reported in the tables.

The Friedman test was used to detect differences within individuals in the caloric intake at dinner across the 3 days of the food records.

The ANOVA and the χ^2^-test were performed to assess the differences among tertiles of dinner caloric intake in the continuous and categorical variables, respectively.

A multiple logistic regression analysis was used to estimate the odds of incident obesity and diabetes, IFG, the metabolic syndrome, and estimated NAFLD, each used as a dependent variable, with tertiles of dinner caloric intakes, used as a dummy variable. Both unadjusted (crude ORs) and adjusted models were carried out for each outcome variable. In the model 1, the following variables were introduced into the model: age (as a continuous variable), sex (males = 1/females = 0), BMI at baseline (as a continuous variable), METS h/week (as a continuous variable), living in a rural area (yes = 1/no = 0); in the model 2, all the above described variables plus intake of total energy and SFA (both as continuous variables), and skipping breakfast (yes = 1/no = 0) were introduced. Since the outcome incidence was evaluated in the living participants at the end of the follow-up during 2008, we were not able to obtain the date of the outcome incidence, and to perform a survival analysis.

The lower tertile of dinner caloric intake (the first tertile) was considered as the reference group, and the other two groups were introduced as dummy variables (STATISTICA software 5.1, Statsoft Italia).

## Results

The mean caloric intake at dinner during the 3 days was 905.4±485.4 kcal; the mean caloric intake from dinner was not significantly different during day 1, 2, and 3 (the latter was the week-end day): 901.1, 896.9, and 918.4 kcal, respectively. Out of 1245 subjects, 103 (8.3%) resulted under-reporters. Among the tertiles of caloric intake at dinner, the percentage of under-reporters did not differ (8.5%, 8.1%, and 8.2% in the first, second, and third tertile, respectively).

Baseline lifestyle and dietary characteristics according to the tertile of percentage of daily caloric intake at dinner are summarized in Table 1; clinical and laboratory values of the participants are reported in Table 2. Subjects in the first tertile were less likely to skip breakfast and consumed lower total energy and SFA, while percentage of calories from carbohydrates, protein, total fat and polyunsaturated fatty acids (PUFA), and fiber and alcohol intake did not differ from the other tertiles (Table 1). The dinner dietary composition, with the exception of total caloric intakes, did not differ among groups (Table 1). At baseline, no significant difference was found among tertiles for age, sex, educational level, drugs used, blood pressure and anthropometric values, laboratory variables, and prevalence of NAFLD, IFG, and the metabolic syndrome (Tables 1–2).

**Table 1:** Baseline lifestyle and dietary characteristics by the percentage of total daily caloric intake from dinner.

	Lower tertile	Middle tertile	Higher tertile
Number	423	418	404
% total kcal from dinner	<33	33–48	≥48
Physical activity (METS h/week)^1^	22.4±9.6	21.2±8.9	21.3±9.1
Actual smoking (%)	24.6	24.2	23.3
Hours sleep/day^1^	7.2±1.1	7.1±1.2	7.1±1.2
Antidepressant use (%)	4.0	2.6	3.5
Restaurant foods >3/week	2.4	2.9	3.9
Skipping breakfast (%)	7.8	28.7^a^	22.3^a^
After-dinner eating (%)	13.2	12.9	13.4
Kcal from after-dinner eating^1^	100.3±15.7	104.0±36.9	105.6±37.9
Frequency of eating occasions^2^			
1	2.4	2.9	2.5
2	14.9	13.9	12.6
3	47.5	48.8	47.3
4	12.3	12.7	13.9
≥5	22.9	21.8	23.8
Total intakes			
Total kcal^1^	2053.9±657.3	2165.5±631.5^b^	2132.7±677.7
Carbohydrates (% kcal)^1^	48.9±7.3	48.8±6.6	48.4±7.4
Protein (% kcal)^1^	16.3±2.9	16.1±2.3	16.2±2.5
Fat (% kcal)^1^	34.7±6.0	34.8±5.6	35.4±6.0
SFA (% kcal)^1^	11.8±3.0	11.9±2.9	12.3±3.7^b^
PUFA (% kcal)^1^	4.3±1.6	4.2±1.5	4.3±1.4
Fiber (g/day)^1^	21.7±10.5	21.0±8.1	21.0±9.8
Alcohol (g/day)^3^	10.0 (30.0)	5.0 (30.0)	10.0 (20.0)
Dinner intakes			
Kcal^1^	522.4±236.3	860.1±284.1^a^	1353.4±477.5^a^
Variance of kcal from dinner	55849.7	80718.3	227985.4
Protein (% kcal)^1^	18.7±3.0	18.6±2.3	18.6±2.6
Fat (% kcal)^1^	35.5±6.0	35.6±5.6	36.2±6.0
Fiber (g/1000 kcal)^1^	4.6±1.8	4.5±1.6	4.6±1.7
Therapy			
Estrogen/steroid drugs (%)	5.9	6.2	6.2
Antidepressant/antipsychotic drugs (%)	4.0	2.6	3.5
Oral hypoglycemic drugs/insulin (%)	0	0	0

1 mean±SD.

2 eating occasion = every episode with ≥15% of the total daily caloric intake.

3 median (inter-quartile range).

a p<0.01 vs tertile 1;

b p<0.05 vs tertile 1; p-values were evaluated by ANOVA or chi-square-test.

Metabolic equivalent of the activity (MET); saturated fatty acids (SFA); polyunsaturated fatty acids (PUFA).

**Table 2:** Baseline clinical and laboratory characteristics by the percentage of total daily caloric intake from dinner.

	Lower tertile	Middle tertile	Higher tertile
Number	423	418	404
Age (years)^1^	54.4±5.7	54.5±5.7	54.1±5.6
Males (%)	47.5	45.0	45.3
Education (%)			
Secondary school	19.4	17.5	19.3
Graduated	9.0	8.4	9.2
Living in a rural area (%)	40.9	40.0	36.9
Systolic blood pressure (mmHg)^1^	131.6±15.4	132.3±15.9	131.0±14.9
Diastolic blood pressure (mmHg)^1^	81.7±8.2	82.6±9.7	81.9±8.8
BMI (kg/m^2^)^1^	24.6±2.9	24.9±2.7	24.9±2.9
Waist circumference (cm)^1^	86.8±10.9	87.6±9.9	87.5±10.6
Fasting glucose (mg/dl)^1^	99.4±20.1	99.5±18.2	101.0±23.8
HbA1c (%)	5.04±0.45	5.07±0.44	5.09±0.47
Triglycerides (mg/dl)^2^	113.0 (63.0)	107.0 (69.0)	113.0 (67.0)
Total cholesterol (mg/dl)^1^	219.4±40.1	213.7±42.0	218.0±40.3
HDL cholesterol (mg/dl)^1^	62.4±14.1	61.2±13.0	61.4±13.5
Fasting insulin (µU/ml)^2^	6.7 (1.8)	6.6 (1.7)	6.7 (2.3)
HOMA-IR score (mmol/l×µU/ml)^2^	1.6 (0.6)	1.6 (0.6)	1.6 (0.7)
ALT (UI/l)^2^	17.0 (12.0)	18.0 (13.0)	17.5 (11.0)
GGT (UI/l)^2^	17.0 (17.0)	17.0 (13.0)	17.0 (16.0)
NAFLD score^2^	−3.0 (1.4)	−2.9 (1.3)	−2.9 (1.4)
Prevalent NAFLD^3^ (%)	10.7	7.5	9.3
CRP (mg/l)^2^	1.1 (1.6)	1.0 (1.6)	1.2 (1.7)
IFG (%)	10.4	13.9	14.9
Metabolic syndrome (%)	27.9	30.9	29.7

1 mean±SD.

2 median (inter-quartile range).

3 NAFLD was defined as >5% estimated liver fat percent [15].

a p<0.01 vs tertile 1;

b p<0.05 vs tertile 1; p-values were evaluated by ANOVA or chi-square-test.

Homeostasis model assessment of insulin resistance (HOMA-IR); alanine aminotransferase (ALT); γ-glutamyl transferase (GGT); non-alcoholic fatty liver disease (NAFLD); C-reactive protein (CRP); impaired fasting glucose (IFG).

Mean follow-up period was 6.1±0.34 years (median 5.8 years). Subjects in the first tertile showed a significantly lower BMI and NAFLD prevalence, and a more favourable NAFLD score (Table 3). The incidence of obesity, diabetes, IFG, NAFLD, and metabolic syndrome were respectively: 101/1245 (8.1%), 30/1245 (2.4%), 286/1245 (23.0%), 229/1245 (18.4%), and 172/1245 (13.8%). The incidence of obesity significantly increased from the first to third tertile; the incidence of metabolic syndrome and estimated NAFLD were significantly higher in the upper tertile (Table 3). Individuals who consumed almost half of their daily caloric intake at dinner were 2-fold more likely to become obese in a multiple logistic regression model, after adjusting for age, sex, BMI at baseline, physical activity, living in a rural area, intake of total energy and SFA, and skipping breakfast (Table 4). In the same model, other variables were significantly associated with the incidence of obesity: the physical activity level expressed as METS h/week (OR = 0.94; 95% CI 0.91–0.97; p<0.001); percentage of SFA intake (OR = 1.10; 95% CI 1.03–1.18; p = 0.006); total energy (OR = 1.06; 95% CI 1.01–1.10; p = 0.005 for each 100 kcal increase); skipping breakfast (OR = 2.13; 95% CI 1.20–3.81; p = 0.01).

**Table 3:** Characteristics at follow-up by the percentage of total daily caloric intake from dinner.

	Lower tertile	Middle tertile	Higher tertile
Number	423	418	404
Systolic blood pressure (mmHg)^1^	132.9±16.1	132.8±17.8	132.4±15.7
Diastolic blood pressure (mmHg)^1^	81.8±8.8	82.5±9.2	81.4±9.2
BMI (kg/m^2^)^1^	24.8±3.1	25.2±3.0^a^	25.5±3.2^b^
Waist circumference (cm)^1^	88.6±10.9	89.2±10.4	89.4±10.5
Fasting glucose (mg/dl)^1^	97.7±21.1	97.4±13.7	99.9±20.3
HbA1c (%)	5.03±0.72	5.03±0.50	5.11±0.71
Triglycerides (mg/dl)^2^	109.0 (66.0)	106.0 (73.0)	113.0 (68.0)
Total cholesterol (mg/dl)^1^	220.7±39.7	217.7±39.3	224.4±41.5
HDL cholesterol (mg/dl)^1^	58.7±15.2	56.6±14.6	58.0±14.7
Fasting insulin (µU/ml)^2^	6.5 (5.3)	7.3 (4.9)	6.8 (5.2)
HOMA-IR score (mmol/l×µU/ml)^2^	1.5 (1.2)	1.7 (1.2)	1.6 (1.2)
ALT (UI/l)^2^	20.0 (11.0)	22.0 (13.0)	22.0 (14.0)
GGT (UI/l)^2^	21.0 (19.0)	21.0 (15.0)	22.0 (21.0)
NAFLD score^2^	−1.7 (1.9)	−1.3 (1.8)	−1.2 (2.0)^a^
Prevalent NAFLD^3^ (%)	20.4	17.8	28.2^b^
CRP (mg/l)^2^	1.0 (1.0)	1.0 (1.7)	1.0 (1.5)
Incident obesity (%)	4.7	8.4^a^	11.4^b^
Incident diabetes (%)	1.7	1.9	3.7
Incident IFG (%)	22.2	22.5	24.3
Incident NAFLD^4^ (%)	16.5	15.0	23.8^a^
Incident metabolic syndrome (%)	11.1	14.4	16.1^a^
Therapy			
Estrogen/steroid drugs (%)	6.1	6.0	6.2
Antidepressant/antipsychotic drugs (%)	4.3	2.9	3.9
Oral hypoglycemic drugs/insulin (%)	1.2	1.4	2.5

1 mean±SD.

2 median (inter-quartile range).

3 NAFLD was defined as >5% estimated liver fat percent [15].

4 incident NAFLD was not equal to the difference between prevalent NAFLD at follow-up minus prevalent NAFLD at baseline, since some individuals whose estimated liver fat percent at baseline was >5%, showed a <5% liver fat percent at follow-up, i.e. their metabolic pattern ameliorated during follow-up.

a p<0.05 vs tertile 1;

b p<0.01 vs tertile 1; p-values were evaluated by ANOVA or chi-square-test.

Homeostasis model assessment of insulin resistance (HOMA-IR); alanine aminotransferase (ALT); γ-glutamyl transferase (GGT); non-alcoholic fatty liver disease (NAFLD); C-reactive protein (CRP); impaired fasting glucose (IFG).

**Table 4:** Association between outcomes at follow-up (dependent variables) and the percentage of total daily caloric intake from dinner in multiple logistic regression models.

	Lower tertile	Middle tertile	Higher tertile
		OR; 95% CI; p	OR; 95% CI; p
Incident obesity (%)			
Crude	1	1.84; 1.04–3.25; 0.03	2.59; 1.50–4.46; <0.001
Model 1^1^	1	1.88; 0.97–3.66; 0.06	2.75; 1.45–5.20; 0.002
Model 2^2^	1	1.79; 0.89–3.62; 0.10	2.33; 1.17–4.65; 0.02
Incident diabetes (%)			
Crude	1	1.16; 0.42–3.23; 0.78	2.29; 0.92–5.69; 0.07
Model 1^1^	1	1.13; 0.40–3.15; 0.82	2.36; 0.94–5.90; 0.07
Model 2^2^	1	0.97; 0.34–2.78; 0.96	2.26; 0.89–5.75; 0.09
Incident IFG (%)			
Crude	1	1.00; 0.98–1.01; 0.76	1.00; 0.99–1.02; 0.55
Model 1^1^	1	1.00; 0.98–1.01; 0.66	1.00; 0.99–1.02; 0.64
Model 2^2^	1	1.00; 0.97–1.01; 0.61	0.99; 0.97–1.01; 0.49
Incident metabolic syndrome (%)			
Crude	1	1.34; 0.89–2.02; 0.16	1.53; 1.02–2.30, 0.04
Model 1^1^	1	1.32; 0.88–2.00; 0.18	1.54; 1.03–2.32; 0.04
Model 2^2^	1	1.32; 0.87–2.01; 0.19	1.52; 1.01–2.30; 0.04
Incident NAFLD (%)			
Crude	1	0.89; 0.61–1.30; 0.56	1.58; 1.12–2.24; 0.01
Model 1^1^	1	0.86; 0.59–1.25; 0.43	1.54; 1.09–2.18; 0.01
Model 2^2^	1	0.88; 0.60–1.30; 0.53	1.56; 1.10–2.22; 0.01

1 Model 1: multiple logistic regression model, after adjustment for age, sex, BMI at baseline, METS h/week, living in a rural area.

2 Model 2: multiple logistic regression model, after adjustment for age, sex, BMI at baseline, METS h/week, living in a rural area, intake of total energy and saturated fat, and skipping breakfast.

Impaired fasting glucose (IFG); non-alcoholic fatty liver disease (NAFLD); metabolic equivalent of the activity (MET).

The incidence of the metabolic syndrome (OR = 1.52; 95% CI 1.01–2.30; p = 0.04) and the estimated NAFLD (OR = 1.56; 95% CI 1.10–2.22; p = 0.01) were significantly higher in the third tertile of daily caloric intake at dinner (Table 4).

The incidence of diabetes was calculated also taking into account the HbA1c criteria (values ≥6.5%) and was 1.2%, 1.9% and 3.2% in the 423, 418 and 404 individuals, respectively. The association between incident diabetes, diagnosed according to the HbA1c criteria, and the second and third tertiles were respectively: OR = 1.44 (95% CI 0.45–4.56; p = 0.54) and OR = 2.78 (95% CI 0.96–8.10; p = 0.06) in the adjusted model (model 2).

The results did not significantly change after excluding the 103 under-reporters or the 164 individuals who consumed foods after-dinner, and after adjusting for total fiber and alcohol intake, dinner nutrient intakes, restaurant food consumption, sleep duration, number of eating occasions, the follow-up period and use of hypoglycemic drugs, insulin, antidepressant/antipsychotic, and estrogen/steroid at follow-up.

## Discussion

In our population-based prospective cohort of middle-aged individuals, energy intake at dinner was significantly associated with the incidence of obesity, metabolic syndrome, and estimated NAFLD. Subjects consuming the largest amount of calories at dinner (“big dinner”) were almost one third of our sample, in line with the literature [12], [14]. After a 6-year follow-up, those individuals were 2-fold more likely to be obese. This association remained significant after adjusting for the intake of energy, SFA and fiber, exercise, skipping breakfast, and other covariates.

### Timing of meals and obesity

A few cross-sectional studies have shown that assuming more of the daily energy intake in the evening is associated with an increased risk of overweight and obesity [12]–[13], [28]–[29] while consuming more of the daily calories at lunch or breakfast [12], [28], [30] are inversely associated. A large prospective cohort study found that both skipping breakfast and eating late at night are related to an increased risk of coronary heart disease. This was intriguing since 76% of late-eaters had breakfast [4]. It has been hypothesized that the timing of the meal may be a proxy for healthy lifestyle habits and/or for consuming specific foods, like high-fat foods at dinner or high-fiber foods at breakfast [4], [31]. Eating in the late evening seems to have a lower satiety value than eating in the morning [31]. Nevertheless, in our cohort, there were no differences among groups for dietary pattern at dinner, as well as exercise, smoking, sleep duration and alcohol intake.

Timing of food intake seem to play a role also in weight loss strategies: late lunch eaters lost less weight than early eaters [32]; overweight/obese women with metabolic syndrome lost significantly more weight after a low-calorie dinner weight-loss program than after an isocaloric high-calorie dinner program [33].

Not all studies confirmed the direct association between weight and food intake at dinner [14], [34]. Yet, a positive association between percentage of evening energy intake and long-term weight change was evident in specific subgroups (smoking men, physically active men, inactive women) in a prospective US cohort [14].

Many hypotheses have been suggested to explain the association between the timing of meals and obesity risk. Insulin sensitivity has been reported to decrease later in the day [35]. Increased levels of free fatty acids, fluctuation in cortisol concentrations, increased urinary epinephrine levels, higher morning ACTH plasma values, and/or a delayed peak in the counteracting activity of glucagon after evening meals may be possible contributors to the circadian modulation of insulin secretion or action [11], [34], [35]–[39]. Diet-induced thermogenesis is significantly higher after the consumption of a snack in the morning than after the consumption of the same snack at night [40], and a reduced evening thermic response may be due to the nocturnal insulin resistance [41]. Habitual nighttime eating or snacking have been associated with reduced fat oxidation, potentially promoting weight gain [42]–[43]. Additionally, morning gastric emptying may be more rapid than evening gastric emptying [44], and an increased efficacy of absorption of dietary carbohydrates has been demonstrated under late suppertime conditions [45]. Circadian variations in satiety hormones, energy expenditure, and genetic mutations of the circadian clock genes have been associated with weight gain and metabolic abnormalities in mice [8]–[10], [46], as well as humans [32], [47].

### Timing of meals and the metabolic syndrome

Eating habits have been associated with the development of insulin resistance and the metabolic syndrome [2]–[3], [48]–[49]. In a small randomized crossover trial, healthy adults showed significantly higher blood values of glucose, insulin, and triglycerides after night meals compared to the ingestion of the same meals during the day [11], thus supporting the well known association between shift work and increased cardiovascular disease and metabolic risk [50]–[51]. These associations seem to be related to the disruption of circadian rhythms leading to worsen the physiological nocturnal decrease of glucose tolerance and adverse metabolic and cardiovascular consequences [35], [52]. Accordingly, in our cohort, individuals consuming a big dinner showed a 1.5-fold higher incidence of metabolic syndrome and a 2-fold higher incidence of diabetes at follow-up; the low number of incident cases of diabetes (30/1245) probably did not allow us to observe a significant associations.

The relationships between caloric intake from dinner and obesity or metabolic syndrome were attenuated after adjusting for breakfast skipping, a condition associated with an increased prevalence of obesity and metabolic diseases [1]–[4], [53]. In our cohort, breakfast skippers more frequently consumed a big dinner and showed a 2-fold higher risk of incident obesity.

Even though eating behaviors are highly inter-related, consuming a big dinner and skipping breakfast were both independently associated with incident obesity and metabolic syndrome in a multiple logistic regression analysis, without reciprocal interference in our cohort.

### Timing of meals and NAFLD

We found that the timing of food intake during the day affected liver fat accumulation and the risk of developing NAFLD, the most common chronic liver disease and an emerging cardio-metabolic risk factor. The exact molecular mechanisms underlying this association are still to be elucidated. A recent metabolomic study discloses extensive and coordinate clock-controlled oscillations of many metabolites, including those within the amino-acid, carbohydrate and lipid metabolic pathways [54]. Other studies are warranted to investigate molecular basis of the interaction between different nutrients and the endogenous metabolic clock in the liver.

### Limitations and strengths

The limitations of a follow-up study are due to competitive risks existing during the follow-up time. We have not evaluated the presence of night eating syndrome; however, we have reduced the possibility of introducing this bias by excluding obese individuals at baseline. Furthermore, the hours of sleep and the percentage of post-dinner eating individuals did not differ among the groups. Our results may be culture-specific and might not be applicable to cultures with different temporal distribution of food intake over the day. Measurement errors are known to be associated with all dietary questionnaires, and underestimation of energy intake seems to be greater in obese individuals [13], [28]. We have excluded obese individuals at baseline, and therefore our prevalence of underreporting was lower than in literature [55]. A 3-day food record may be inadequate to evaluate the individual usual eating patterns. However, it is usually employed to characterize dietary habits of groups of individuals [55]. Furthermore, the comparability of our data with other Italian data [55] is reassuring, although it is not sufficient to establish the validity of the data. The possibility of residual confounding factors cannot be excluded, due to the observational study design. Finally, our results cannot be extended to obese individuals.

This was a prospective study providing extensive and complete clinical measures as well as data on different lifestyle conditions with which to explore confounders and mediators of the associations under investigation.

### Conclusions

A better understanding of the mechanisms of weight gain could have important implications for developing new strategy to counteract the obesity epidemic. The timing of energy intake is a modifiable lifestyle habit that might impact on the incidence of obesity, metabolic syndrome, and NAFLD. Intervention trials are needed to evaluate the opportunity to include in dietary recommendations advice on the time-of-day for food consumption, besides advice on food quality and quantity.
